# Pharmacological differences between human and mouse P2X4 receptor explored using old and new tools

**DOI:** 10.1007/s11302-024-10018-x

**Published:** 2024-05-20

**Authors:** Anna Fortuny-Gomez, Samuel J. Fountain

**Affiliations:** School of Biological Sciences, University of East Anglia, Norwich Research Park, Norwich, UK

**Keywords:** P2X4, Pharmacology, Species difference, Human, Mouse

## Abstract

There is growing interest in the P2X4 receptor as a therapeutic target for several cardiovascular, inflammatory and neurological conditions. Key to exploring the physiological and pathophysiological roles of P2X4 is access to selective compounds to probe function in cells, tissues and animal models. There has been a recent growth in selective antagonists for P2X4, though agonist selectivity is less well studied. As there are some known pharmacological differences between P2X receptors from different species, it is important to understand these differences when designing a pharmacological strategy to probe P2X4 function in human tissue and mouse models. Here, we provide a systematic comparison of agonist and antagonist pharmacology in 1321N1 cells expressing either human or mouse P2X4 orthologues. We identify a rank order of agonist potency of ATP > 2-MeSATP > αβmeATP = BzATP > CTP = γ-[(propargyl)-imido]-ATP for human P2X4 and ATP > 2-MeSATP = CTP > ATPγS = γ-[(propargyl)-imido]-ATP = BzATP for mouse. Human P2X4 is not activated by ATPγS but can be activated by αβmeATP. We identify a rank order of antagonist potency of BAY-1797 = PSB-12062 = BX-430 > 5-BDBD > TNP-ATP = PPADS for human P2X4 and BAY-1797 > PSB-12062 = PPADS > TNP-ATP for mouse. Mouse P2X4 is not antagonised by 5-BDBD or BX-430. The study reveals key pharmacological differences between human and mouse P2X4, highlighting caution when selecting tools for comparative studies between human and mouse and ascribing cellular responses of some commonly used agonists to P2X4.

## Introduction

P2X receptors are a family of ligand-gated ion channels activated by extracellular adenosine 5′-triphosphate (ATP). P2X receptors are formed by the trimerisation of subunits which form a central non-selective cation pore upon channel opening and inter-subunit binding sites for ATP [1, 2]. Open channels are chiefly permeable to Na^+^, K^+^ and Ca^2+^ under physiological conditions, causing membrane depolarisation and increased cytosolic Ca^2+^. Mammalian genomes encode seven P2X receptor subunits (P2X1-7) that can assemble as homomeric and heteromeric receptors dependent upon subtype. There is a resurgent interest in P2X receptors as therapeutic targets following the approval of MK-7264 (gefapixant), a P2X3 receptor antagonist, for refractory or unexplained chronic cough [3, 4]. P2X have established roles in the cardiovascular system including blood pressure control [5] and vascular tone [6, 7]. P2X4 is a promising therapeutic target with studies identifying its role in leukocyte function and inflammation [8–10], microglia function and neuropathic pain [11, 12], pulmonary secretion [13], fluid shear stress responses in vascular endothelium [14] and blood pressure [15]. Early studies relied on broad-spectrum and non-selective antagonists, such as suramin, PPADS and TNP-ATP. PPADS has been shown to inhibit the human P2X4 receptor fully, whilst suramin appears to have non-specific effects at high concentrations [16–18]. Recent advances in the development of P2X4 receptor-selective antagonists include a benzodiazepine derivative called 5-BDBD, the N-substituted phenoxazine derivative PSB-12062 and the phenylurea derivative BX-430 [19–21]. The most recent ones, BAY-1797 (N-[4-(3-chlorophenoxy)-3-sulfamoylphenyl]-2-phenylacetamide) and NC-2600 (developed by Nippon Chemiphar) are orally active and display antinociceptive and anti-inflammatory effects [22–24]. Besides, the completion of phase I clinical trials for NC-2600 as a P2X4 receptor antagonist for the treatment of chronic cough and neuropathic pain seems promising [23, 25]. These recent advances in the development of small molecules [26, 27] and biologics [28] targeting the P2X4 receptor have allowed a better understanding of where drug-like molecules bind to P2X4 receptors [29]. Mouse models continue to be an important tool in the pre-clinical drug development of P2X receptor modulators [4]. Many studies have used molecules presumed to target P2X4 in vivo in mouse models to either validate small molecules or identify physiological roles for P2X4. However, known pharmacological differences between P2X4 receptor orthologues [17], which can occur due to single amino acid differences [30], should draw attention to the usefulness of small molecules in determining the physiological or pathophysiological roles of P2X4 in mouse models if the activity against mouse P2X4 is not directly determined. This is the case for many commercially available P2X4 receptor agonists and antagonists. As it is difficult to fully appraise the selectivity of small molecules at human and mouse P2X4 across different studies and varying techniques employed within them, we have undertaken a systematic pharmacological comparison of commercially available agonists and antagonists at human and mouse P2X4, revealing important pharmacological differences.

## Materials and methods

### Compounds

ATP (≥ 99% purity; Abcam), γ-[(propargyl)-imido]-ATP (≥ 95% purity; Sigma), CTP (≥ 95% purity; Sigma), 2-MeSATP (≥ 98% purity; Tocris), BzATP (≥ 93% purity; Sigma), ATPγS (≥ 90% purity; Tocris), Ap4A (≥ 95% purity; Sigma), suramin (≥ 98% purity; Sigma), PPADS (≥ 98% purity; Sigma) and TNP-ATP (≥ 95% purity; Tocris) were all dissolved in water. 5-BDBD (≥ 99% purity; Tocris), BX-430 (≥ 99% purity; Tocris), PSB-12062 (≥ 98% purity; Sigma) and BAY-1797 (≥ 98% purity; Cambridge Biosciences) were dissolved in dimethyl sulfoxide (DMSO).

### Cells and culture

1321N1 human astrocytoma cells stably expressing human or mouse P2X4 were cultured in Dulbecco’s Modified Eagle Medium containing glucose (4.5 g/L), 2 mM l-glutamine, 10% (v/v) foetal bovine serum, 50 U/mL penicillin and 50 µg/mL streptomycin. Cells were cultured in a humidified incubator at 37 °C with 5% CO_2_. Human P2X4 1321N1 cells have been previously described [31] and express sequence NP_002551. Mouse P2X4 cells express protein sequence NP_035156.

### Intracellular *Ca*^*2*+^ assays

Cells were seeded at a density of 25,000 cells/well in 96-well plates. Assays were performed in SBS buffer containing (mM): 130 mM NaCl, 5 mM KCl, 1.2 mM MgCl_2_, 1.5 mM CaCl_2_, 8 mM D-( +)-glucose and 10 mM HEPES. The solution was pH 7.4, and osmolarity 300 mOsm. Cells were then loaded with Fura-2 loading buffer consisting of SBS supplemented with 0.01% [w/v] of pluronic acid F-127 and 2 µg/mL of Fura-2 AM (Abcam, Cambridge, UK) for 1 h at 37 °C whilst protected from the light. When applicable, cells were incubated with antagonists or vehicle control for 30 min at 37 °C before starting the assay. Finally, cells were placed in a FlexStation 3 microplate reader (Molecular Devices, UK), which recorded the dual-excitation (340 nm and 380 nm) single-emission (510 nm) fluorescence ratio for the Fura-2 dye. The fluorescence measurement at 510 nm with two excitation wavelengths (340 nm for calcium-bound states and 380 nm for calcium-free states) allowed us to quantify and represent the change in intracellular calcium levels as a fluorescence ratio, F ratio (340/380). Readings were taken every 3 s over 250 s. After 20 s of baseline, cells were challenged with agonists administered automatically by the FlexStation 3 device. All experiments were performed at 37 °C.

### Data analysis

Concentration–response curves, where the peak calcium responses, were plotted against the common logarithm (Log10) of each concentration tested and were individually fitted using a modified Hill equation (Hill1 function on the OriginPro software) as outlined below:

$$Y=START+\left(END-START\right)\frac{{x}^{n}}{{k}^{n}+{x}^{n}}$$

where *k* represents the Michaelis constant and *n* is the number of cooperative sites. The EC_50_ (half maximal effective concentration) and IC_50_ (half maximal inhibitory concentration) values were obtained according to the fitted curve and were equal to the *k* value in the Hill1 equation.

All data and statistical analysis were performed using Excel (Microsoft Corporation) and OriginPro software (OriginLab version 9.95, UK). Data distribution was tested using a Shapiro–Wilk test for normality of the mean and Levene’s test for equality of variances. Data that followed a parametric distribution were analysed with two-tailed student’s *t*-tests. Non-parametric datasets were assessed using Mann–Whitney tests. The threshold for statistical significance was considered for *P* values lower than 0.05 throughout (*P < 0.05, **P < 0.01, ***P < 0.001). Data were expressed as mean ± SEM. All experiments were performed in triplicates (technical repeats within one experiment) and repeated three to five times, as indicated by the “N” number of biological repeats.

## Results

### Agonists

**Fig. 1:**
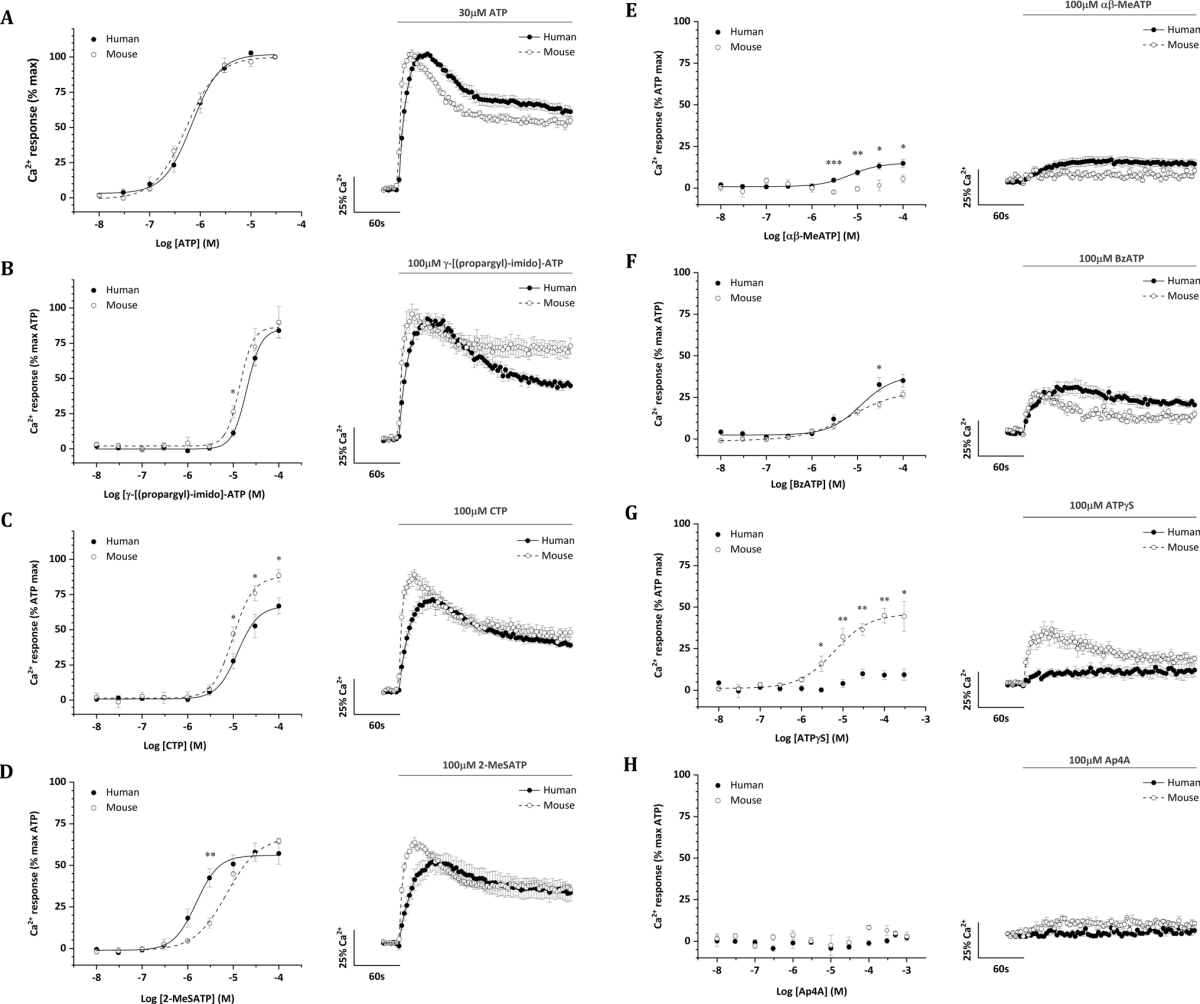
Comparison of agonist ligand effects in 1321N1 cells stably expressing human or mouse P2X4 receptors. Effects of putative agonist ligands **A** ATP (*N* = 7), **B** γ-[(propargyl)-imido]-ATP (*N* = 5), **C** CTP (*N* = 5–7), **D** 2-MeSATP (*N* = 5–7), αβ-MeATP (*N* = 5–7; **E**), BzATP (*N* = 5; *F*); ATPγS (*N* = *5*; **G**) and Ap4A (*N* = 3; **H**). (*Left panels*) Concentration–response curves for human and mouse P2X4. All responses are normalised to the Ca^2+^ response evoked by 30 µM ATP. (*Right panels*) Average evoked Ca^2+^ response at maximal agonist concentrations tested (*N* = 5–7). Data were represented as mean ± SEM. *P < 0.05, **P < 0.01, ***P < 0.001 for evoked Ca^2+^ response human versus mouse at given concentration

ATP was an equipotent agonist at human and mouse P2X4 with EC50 values of 747 ± 180 nM and 565 ± 85 nM, respectively (Fig. 1A). ATP EC80 values for human and mouse were 1.5 ± 0.3 µM and 1.4 ± 0.3 µM, respectively. 1.5 µM ATP was therefore selected to test the effects of antagonists in experiments described later. γ-[(Propargyl)-imido]-ATP activated human and mouse P2X4 (Fig. 1B) but was significantly less potent than ATP at both receptors (Table 1). γ-[(Propargyl)-imido]-ATP activated human and mouse P2X4 with EC50 values of 20 ± 2 µM and 20 ± 5 µM, respectively. Whilst γ-[(propargyl)-imido]-ATP acted as a full agonist at mouse P2X4 (Table 1), the maximal response at human P2X4 was significantly smaller indicating partial agonism (Table 1). The action of CTP also varied between human and mouse P2X4 (Fig. 1C). CTP activated human and mouse P2X4 with EC50 values of 20 ± 4 µM and 10 ± 1 µM, respectively, significantly less potent than the action of ATP at both receptors (Table 1). CTP activated as a full agonist at mouse P2X4 but was a partial agonist at human P2X4 (Table 1), with the maximal response of 67% of ATP. 2-MeSATP was a partial agonist at both human and mouse P2X4 (Fig. 1D), producing a response of 57% and 65% of ATP, respectively (Table 1). 2-MeSATP was had a slightly higher but significant difference in potency at human P2X4 compared to mouse, with EC50 values of 2 ± 0.2 µM and 8 ± 2 µM, respectively. αβmeATP had no significant effect on mouse P2X4 and elicited a small response, 15% maximal compared to ATP, at human P2X4 (Fig. 1E). Though small, the action of αβmeATP at human P2X4 was relatively potent with an EC50 value of 7 ± 0.7 µM, but significantly less potent than ATP (Table 1). BzATP was also a relatively potent agonist at human and mouse P2X4, with EC50 values of 11 ± 2 µM and 24 ± 16 µM, respectively (Fig. 1F). BzATP was a partial agonist at both human and mouse receptors, eliciting a response of 35% and 27% of ATP (Table 1). ATPγS did not activate human P2X4 (Fig. 1G) but acted as a partial agonist at mouse P2X4 with an EC50 value of 18 ± 1 µM. Ap4A was tested up to 1 mM and did not activate either human or mouse P2X4 receptors (Fig. 1H). These data reveal a rank order potency of ATP > 2-MeSATP >  αβmeATP = BzATP > CTP = γ-[(propargyl)-imido]-ATP for human P2X4 and ATP > 2-MeSATP = CTP > ATPγS = γ-[(propargyl)-imido]-ATP = BzATP. Properties and comparisons of agonist action between human and mouse receptor orthologues are summarised in Table 1.

**Table 1:** Pharmacological properties of agonists at human and mouse P2X4

Ligand	EC_50_		Efficacy^#^	
	Human	Mouse	Human	Mouse
ATP	747 ± 180 nM	565 ± 85 nM	100%	100%
γ-[(Propargyl)-imido]-ATP	20 ± 2 µM	20 ± 2 µM	85 ± 5%*	90 ± 11%
CTP	20 ± 4 µM	10 ± 1 µM	67 ± 6%*	89 ± 5%
2-MeSATP	2 ± 0.2 µM	8 ± 2 µM**	57 ± 6%*	65 ± 2%
α,β-meATP	7 ± 0.7 µM	ND	15 ± 2%*	5 ± 2%*
BzATP	11 ± 2 µM	24 ± 16 µM	35 ± 4%*	27 ± 2%*
ATPγS	ND	18 ± 1 µM	9 ± 3%*	45 ± 4%*

*ND* not determined

^*^Significantly less than 100% ATP response implies partial agonist

^**^P < 0.05 human *vs* mouse EC_50_

^**#**^Maximal response to ligand as a percentage of maximal response to ATP (30 µM)

### Antagonists

**Fig. 2:**
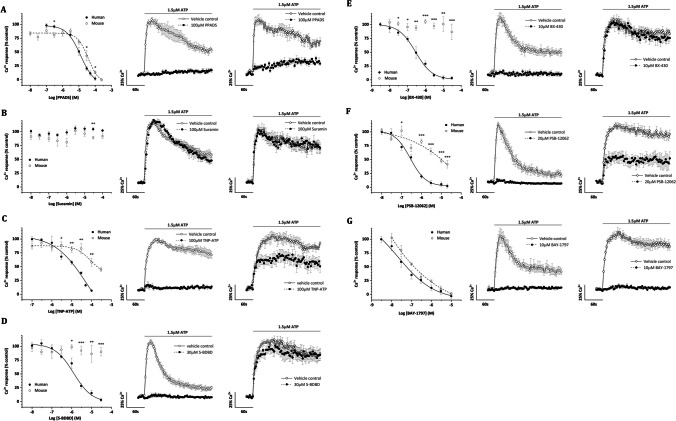
Comparison of antagonist ligand effects in 1321N1 cells stably expressing human or mouse P2X4 receptors. Effects of putative antagonist ligands tested against Ca^2+^ responses evoked by EC_80_ ATP (1.5 µM). **A** PPADS (*N* = 5), **B** suramin (*N* = 5), **C** TNP-ATP (*N* = 5), **D** 5-BDBD (*N* = 5), **E** BX-430 (*N* = 5), **F** PSB-12062 (*N* = 5), **G** BAY-1797 (*N* = 5). (*Left panels*) Concentration–response curves for human and mouse P2X4. Average evoked Ca^2+^ response at maximal antagonist concentrations tested (*N* = 5) for human (*central panels*) and mouse (*right panels*) P2X4. Data were represented as mean ± SEM. *P < 0.05, **P < 0.01, ***P < 0.001 for evoked Ca^2+^ response human versus mouse at given concentration

**Fig. 3:**
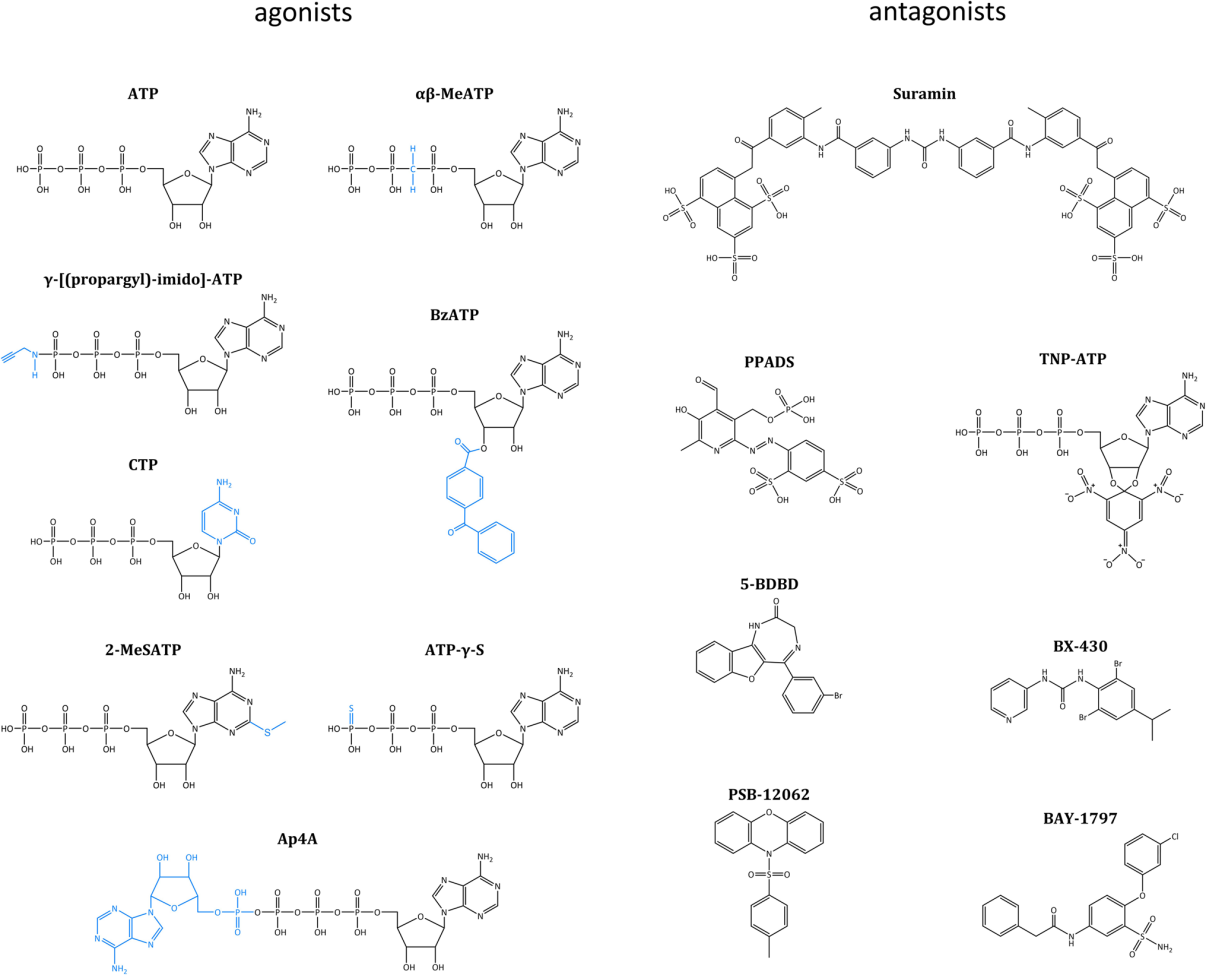
Chemical structures of agonists are shown with blue to highlight the structural differences with the endogenous ligand ATP

The broad-spectrum purinergic receptor antagonist PPADS inhibited human and mouse P2X4 equipotently, with IC50 values of 34 ± 16 µM and 42 ± 14 µM, respectively (Fig. 2A). However, both receptors were completely insensitive to suramin up to 100 µM (Fig. 2B). TNP-ATP displayed selectivity for human over mouse P2X4, with IC50 values of 17 ± 5 µM and 93 ± 4 µM, respectively (Fig. 2C). Whilst TNP-ATP completely inhibited human P2X4, the mouse P2X4 responses were only inhibited by 43% (*P* < 0.01 *vs* human; *N* = 5) at 100 µM (Table 2). 5-BDBD displayed very good selectivity between P2X4 orthologues (Table 2), with mouse P2X4 being insensitive to 5-BDBD up to 100 µM (Fig. 2D). 5-BDBD was a relatively potent antagonist at human P2X4 with an IC50 value of 1 ± 0.3 µM and could completely inhibit receptor activity (Fig. 2D). BX-430 also displayed excellent selectivity for human over mouse P2X4, inhibiting human P2X4 with an IC50 of 426 ± 162 nM and abolished receptor activity (Fig. 2E). Mouse P2X4 was insensitive to BX-430 tested up to 100 µM. Compared to 5-BDBD and BX-430, PSB-12062 displayed modest selectivity for human over mouse P2X4 and inhibited activity with an IC50 of 248 ± 41 nM and 3 ± 2 µM, respectively (Fig. 2F). Whilst PSB-12062 abolished activity at human P2X4, activity at mouse P2X4 was only inhibited by 59% at the maximum concentration of 20 µM tested (Fig. 2F). Finally, we investigated BAY-1797, a more recently developed compound. BAY-1797 did not display P2X4 orthologue selectivity, equipotently inhibiting human and mouse P2X4 with an IC50 of 210 ± 74 nM and 141 ± 24 nM, respectively (Fig. 2G). These data reveal a rank order potency of BAY-1797 = PSB-12062 = BX-430 > 5-BDBD > TNP-ATP = PPADS for human P2X4 and BAY-1797 > PSB-12062 = PPADS > TNP-ATP for mouse P2X4 (Table 2). Properties and comparisons of antagonist action between human and mouse receptor orthologues are summarised in Table 2. The chemical structures of all ligands investigated are shown in Fig. 3.

**Table 2:** Pharmacological properties of antagonists at human and mouse P2X4

Ligand	IC_50_		% inhibition	
	Human	Mouse	Human	Mouse
PPADS	34 ± 16 µM	42 ± 14 µM	98 ± 3% (100 µM)	87 ± 16%(100 µM)
TNP-ATP	17 ± 5 µM	93 ± 4 µM^*^	94 ± 1% (100 µM)	43 ± 7% (100 µM)*
5-BDBD	1 ± 0.3 µM	ND	97 ± 3% (30 µM)	10 ± 8% (30 µM)*
BX-430	426 ± 162 nM	ND	96 ± 3% (10 µM)	0 ± 3% (10 µM)*
PSB-12062	248 ± 41 nM	3 ± 2 µM	99 ± 4% (20 µM)	59 ± 7% (20 µM)*
BAY-1797	210 ± 74 nM	141 ± 74 nM	100 ± 4% (10 µM)	99 ± 2% (10 µM)

*ND* not determined

^*^P < 0.05 human *vs* mouse

## Discussion

Our study reveals clear pharmacological differences between human and mouse P2X4 receptor orthologues, particularly antagonist pharmacology. This information should be informative when selecting antagonists to study the roles of P2X4 in mouse models and mouse-derived cells. The study also highlights the importance of confirming antagonist activity at the mouse P2X4 receptor when designing mouse in vivo studies. BzATP is often purported as a selective P2X7 agonist, though our data suggests it is a partial agonist at both human and mouse P2X4. Though BzATP is a full agonist at P2X7, it is active at P2X4 in the same micromolar range [28]. This is therefore an important consideration when applying BzATP as a tool to probe P2X7 function in cells and tissues, and such experiments need to be supported by molecular work or selective antagonism of P2X7. Likewise, αβmeATP often purported as a selective P2X1 and P2X3 agonist in the literature acts as a weak partial agonist of human P2X4. Caution should therefore be applied by using either BzATP or αβmeATP when using intracellular Ca^2+^ to ascribe P2X receptor subtype function in cells and tissues.

Our study identifies γ-[(propargyl)-imido]-ATP as a novel P2X4 partial agonist. The partial agonist is equipotent at human and mouse P2X4, but significantly less potent than ATP. The reduction in potency may represent an alteration in bonding between ATP phosphate moieties, particularly the terminal gamma phosphate which plays a critical role in bonding with key positively charged amino acid residues within the ATP binding pocket [2]. ATPγS is a non-hydrolysable ATP analogue. In our study, we evidence that ATPγS up to 300 µM does not activate human P2X4. However, studies using human cells have attributed effects of ATPγS to the activation of P2X4 [32]. It is possible therefore that P2X4 contributes to the indirect effects of ATPγS, as our data does not support a direct agonist effect on human P2X4. Our results differ from the findings of Bianchi et al. [33] who demonstrate partial agonism of the human P2X4 receptor by ATPγS. Recently, the inhibitory effect of ATPγS on gamma oscillations in mouse brain was investigated [34]. Here, the effect of ATPγS was reversed by PSB-12062, and more importantly, lost in P2X4 knockout mice. Our data support the ability of ATPγS in activating mouse P2X4, albeit as a partial agonist. There has been mixed evidence in the literature regarding the sensitivity of human and mouse P2X4 receptors to Ap4A. Our data with Ap4A differ from those of Abdelrahman et al. [35] and Jones et al. [17] in which both studies demonstrate activation of human P2X4 by Ap4A at nanomolar concentrations. Abdelrahman et al. [35] suggest mouse P2X4 is insensitive to Ap4A, in agreement with our current study, yet Jones et al. [17] evidence that Ap4A activates mouse P2X4 at low micromolar concentrations. We currently cannot fully explain these differences. Interestingly, previous studies suggest Ap4A is equipotent with ATP at rat P2X4 [36].

Previous studies have identified sensitivity of rat P2X4 to 5-BDBD [37], yet despite the lack of activity at mouse P2X4, 5-BDBD has been employed in several mouse studies to infer a physiological or pathophysiological role for P2X4 including arthritis [38], intracerebral haemorrhage [39], airway inflammation [40], bladder voiding [41], T-cell recruitment [42] and cancer [43]. Our study would suggest that any effects of 5-BDBD in such studies are not due to homomeric P2X4, assuming the molecular composition of P2X4 used in our study is a faithful surrogate of the native P2X4 receptor in mice. Our study finds that Ap4A is not an agonist at mouse P2X4 and 5-BDBD is not an antagonist at mouse P2X4. These findings differ from the work of Abdelrahman et al. [35] where mouse P2X4 stably expressed in 1321N1 cells is also the model used. Though is not clear from this study the isoform of mouse P2X4 used to generate the stable cells, we assume the sequence is the same as given by accession number Q9JJX6 as this is discussed later in the manuscript. This variant of mouse P2X4 is shorter than the variant expressed in our study (NP_035156), with a gap of 27 amino acids in the ectodomain of the receptor. The use of different mouse P2X4 receptor variants in the two studies may very well explain the pharmacological differences reported. Importantly, this also suggests that variants of mouse P2X4 are pharmacologically distinct. Further investigation is required to systematically test this and understand how tissue expression of P2X4 variants affects the sensitivity of P2X4 agonists and antagonists. In addition, our data demonstrates clear pharmacological differences between human and mouse P2X4 pharmacology. Combining this information with the work of others also illustrates major pharmacological differences for both agonists and antagonists between mouse and rat P2X4, including Ap4A [36], 5-BDBD [37] and PPADS [30].

Our study highlights the importance of understanding pharmacological differences between P2X4 receptor orthologues when selecting tools to investigate P2X4 function in cells, tissues, and in vivo. Our study also raises the possibility that expression of homomeric P2X4 receptors in cell lines may not be faithful surrogates of native P2X4 channels, with the possibility of heteromeric assembles and association with auxiliary subunits altering pharmacological properties, as observed for other ion channel families. This warrants further investigation.

## Data Availability

Data presented with the manuscript are available on request from the corresponding author.

## Abbreviations

CTP: Cytidine 5′-triphosphate
AP4A: Diadenosine tetraphosphate
2-MeSATP: 2-(Methylthio)adenosine 5′-triphosphate
αβ-MeATP: α,β-Methyleneadenosine 5′-triphosphate
BzATP: 2′(3′)-O-(4-Benzoylbenzoyl)adenosine 5′-triphosphate
γ-imidoATP: Adenosine-5′-[γ-(propargyl)-imido]triphosphate
ATPγS: Adenosine-5′-O-(3-thio-triphosphate)
Suramin: 8,8′-{Carbonylbis[imino-3,1-phenylenecarbonylimino(4-methyl-3,1-phenylene)carbonylimino]}di(1,3,5-naphthalenetrisulfonic acid)
PPADS: Pyridoxalphosphate-6-azophenyl-2′,4′-disulfonic acid
TNP-ATP: 2′,3′-O-(2,4,6-Trinitrophenyl)-ATP
5-BDBD: 5-(3-Bromophenyl)-1,3-dihydro-2H-benzofuro[3,2-e]-1,4-diazepin-2-one
PSB-12062: N-(p-methylphenylsulfonyl)phenoxazine
BX-430: 1-(2,6-Dibromo-4-isopropyl-phenyl)-3-(3-pyridyl)urea
BAY-1797: N-[4-(3-Chlorophenoxy)-3-sulfamoylphenyl]-2-phenylacetamide
